# Supplementary material for: Short-Term Outcomes after D2 Gastrectomy with Complete Mesogastric Excision in Patients with Locally Advanced Gastric Cancer: A Systematic Review and Meta-Analysis of High-Quality Studies
Source: Cancers (Basel). 2023 Dec 31;16(1):199. doi: 10.3390/cancers16010199 (PMC10778561; doi:10.3390/cancers16010199)
Supplement: Supplementary file 1 [file cancers-16-00199-s001.zip › Supplementary materials.pdf]

# ***Short-Term Outcomes after D2 Gastrectomy with Complete Mesogastric Excision in Patients with Locally Advanced Gastric Cancer: A Systematic Review and Meta-Analysis of High-Quality Studies***

SUPPLEMENTARY MATERIALS

Index


| | |
|---|---|
| 1) Search queries | 2 |
| 2) Funnel plots of subgroup analysis and bubble plots of metaregression | 3 |
| 3) Risk of bias assessment: traffic light plot | 5 |
| 4) Secondary endpoints: Funnel plots of publication bias | 7 |

1) **Search queries**

**Pubmed and Cochrane Library = 90 results**

*((gastric cancer) OR (gastric neopl*) OR (gastric adenocarcinoma) OR (cancer of the stomach) OR (adenocarcinoma of the stomach) OR (gastric tumor)) AND ((complete mesogastr* excision) OR (mesogastr*) OR (CME) OR (enjoyable space) OR (holy plane))*

**Scopus = 663 results**

*("gastric cancer" OR "gastric neopl*" OR "gastric adenocarcinoma" OR "cancer of the stomach" OR "adenocarcinoma of the stomach" OR "gastric tumor") AND ("complete mesogastr* excision" OR "mesogastr*" OR "CME" OR "enjoyable space" OR "holy plane")*

## 2) Funnel plots of subgroup analysis and bubble plots of metaregression

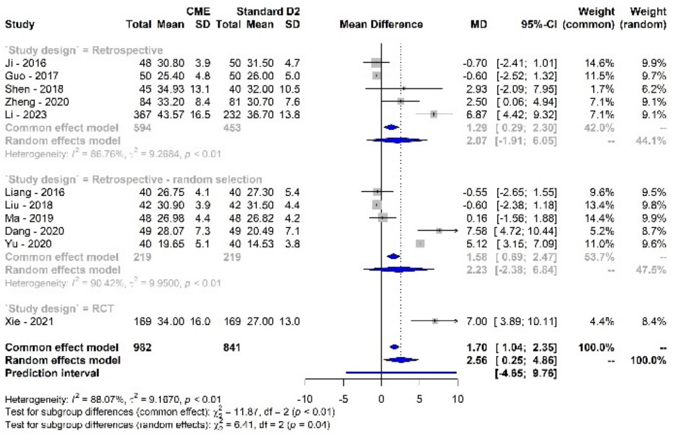


*Subgroup analysis: study design*

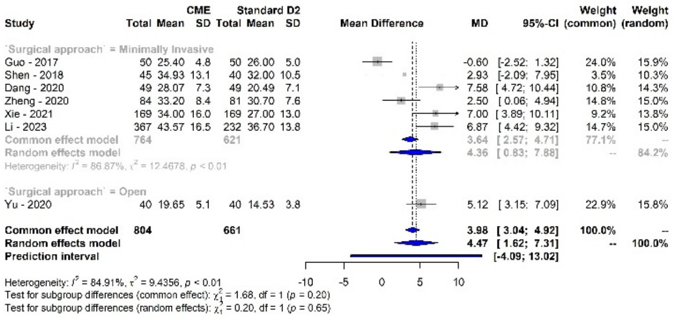


*Subgroup analysis: surgical approach*

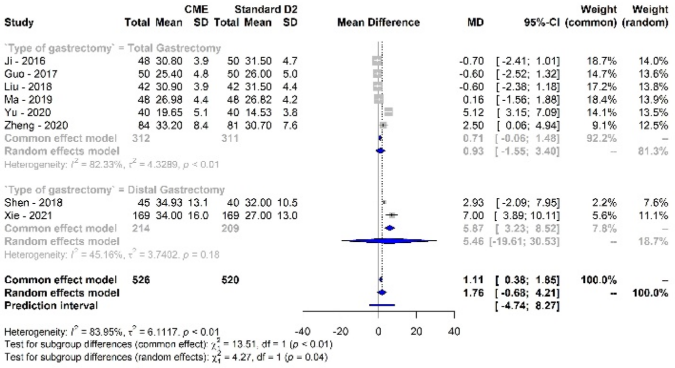


*Subgroup analysis: type of gastrectomy*

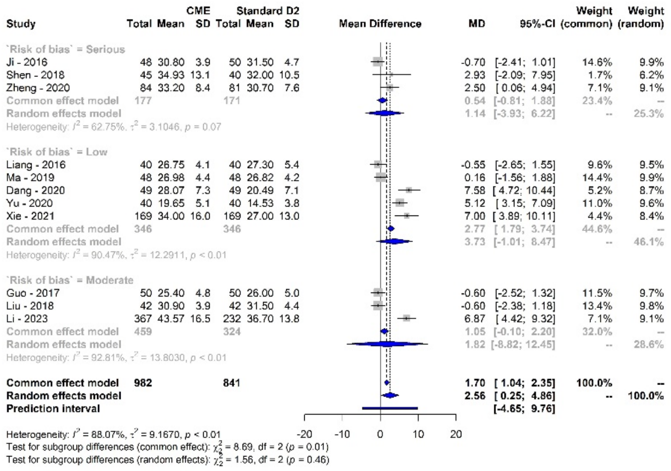


*Subgroup analysis: risk of bias*

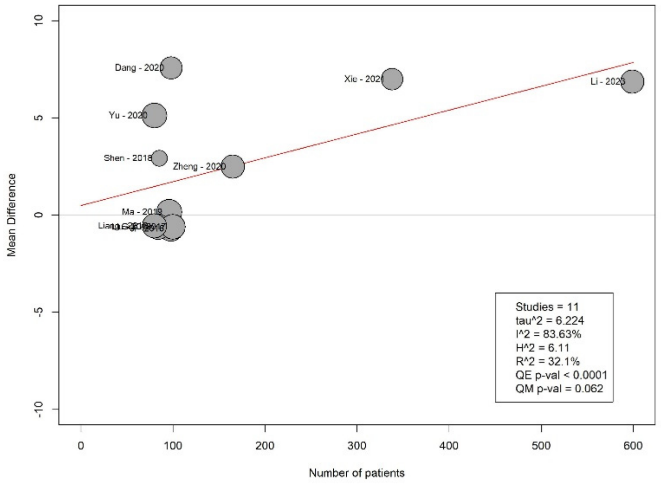


*Metaregression analysis: number of patients per study*

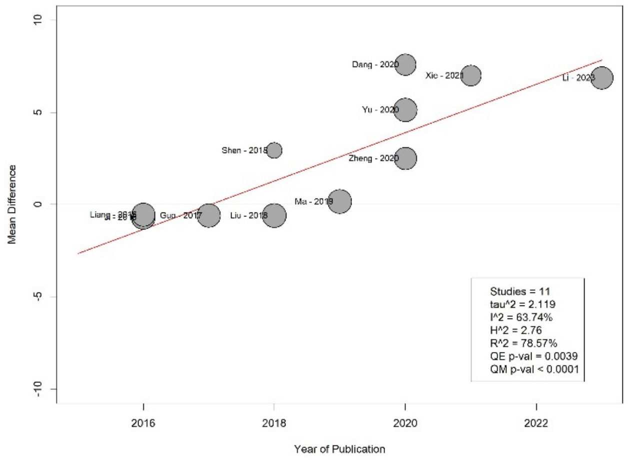


*Metaregression analysis: year of publication*

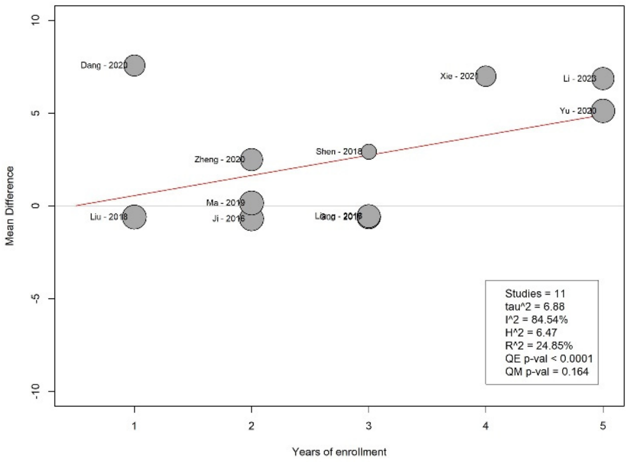


*Metaregression analysis: years of enrollment*

## 3) Risk of bias assessment: traffic light plot

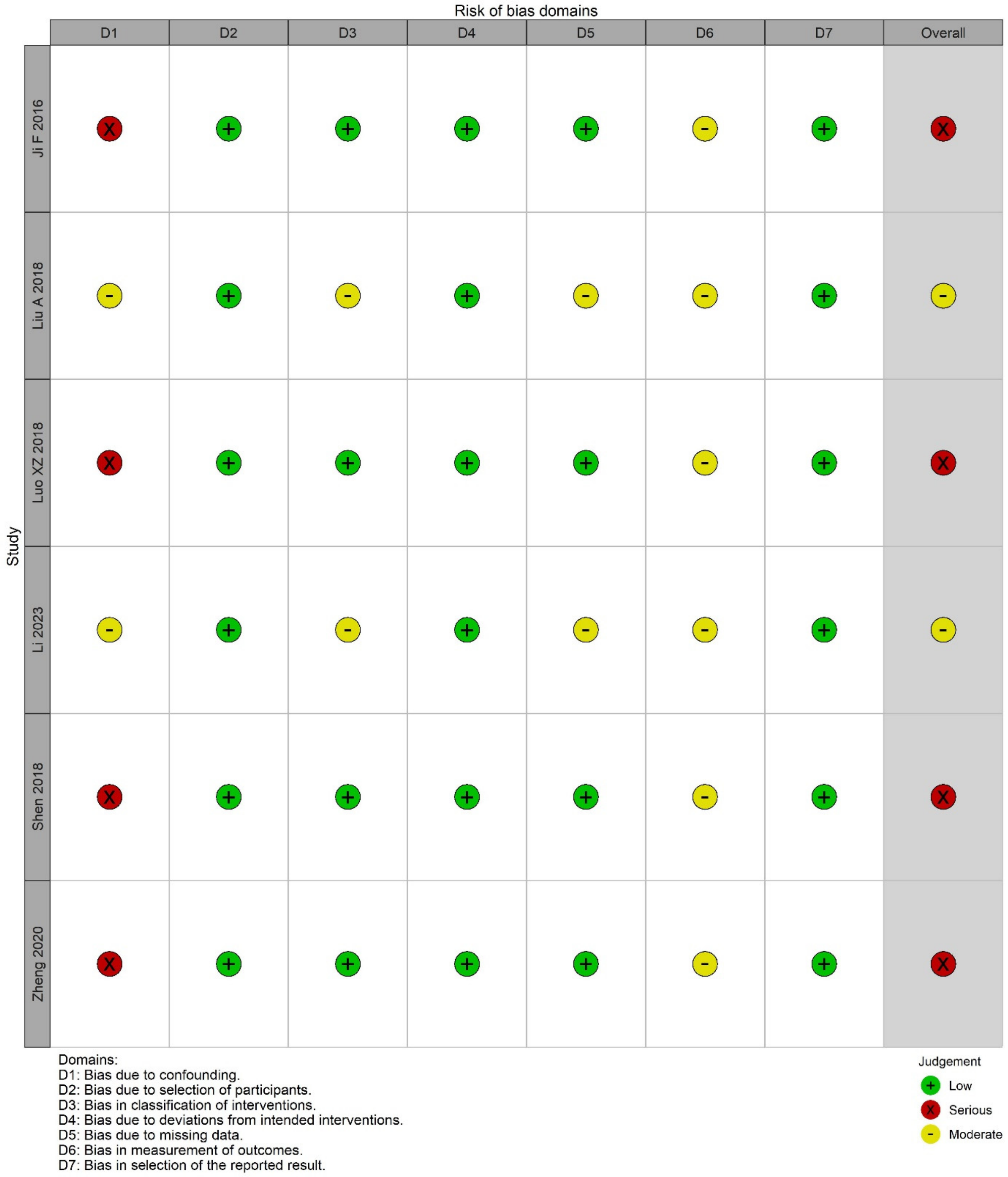


*Risk of bias assessment for non-randomized studies - traffic light plot*

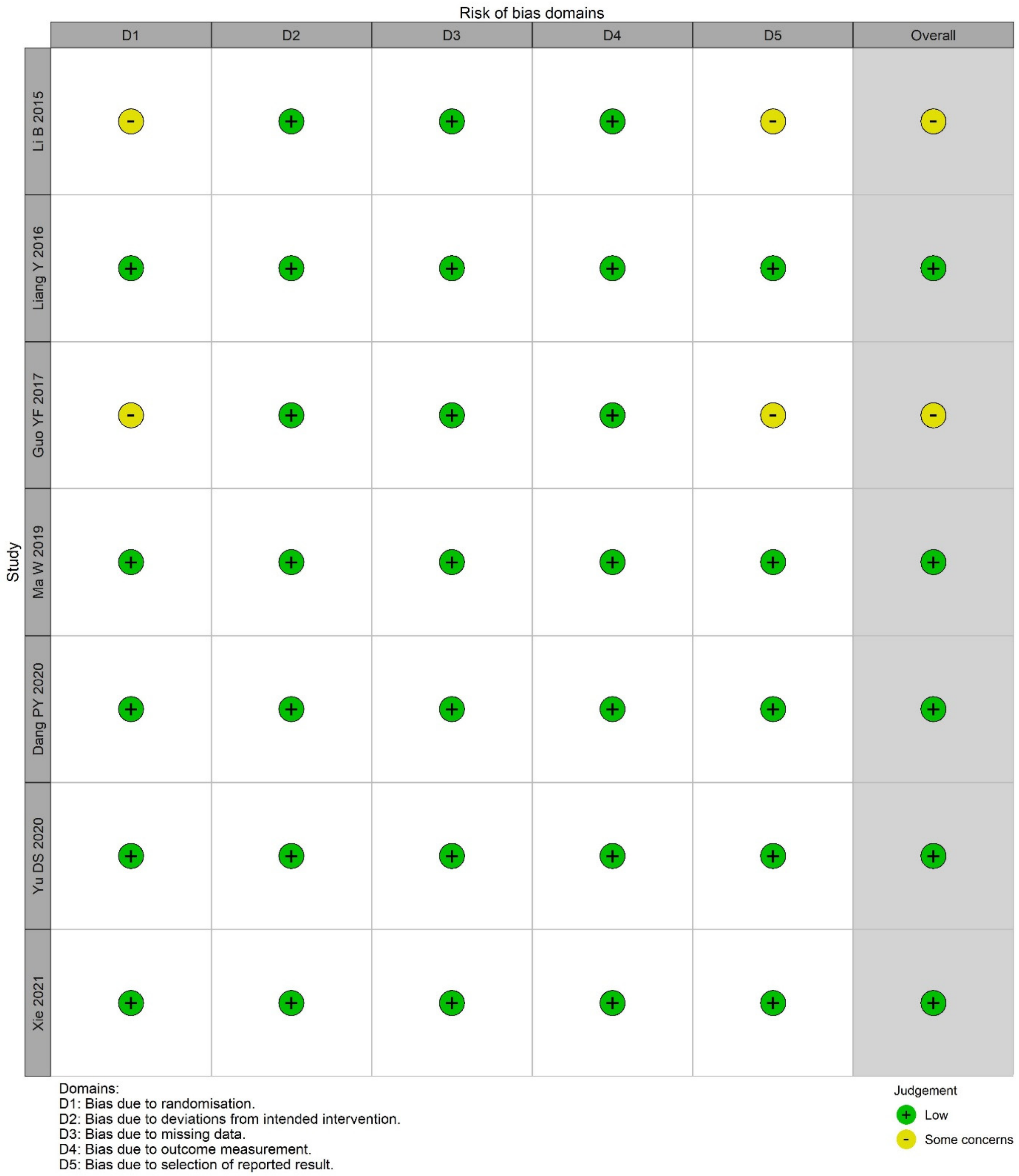


*Risk of bias assessment for randomized studies - traffic light plot*

## 4) Secondary endpoints: Funnel plots of publication bias

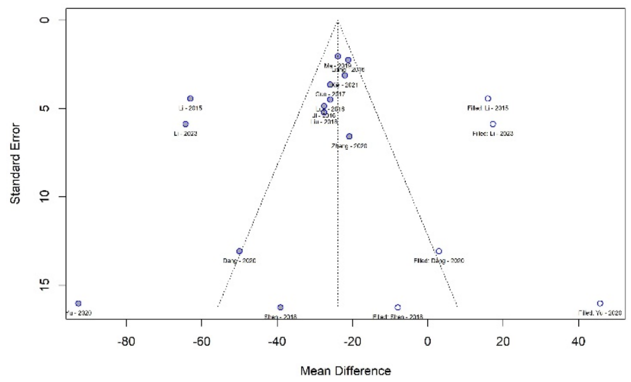


*Intraoperative blood loss*

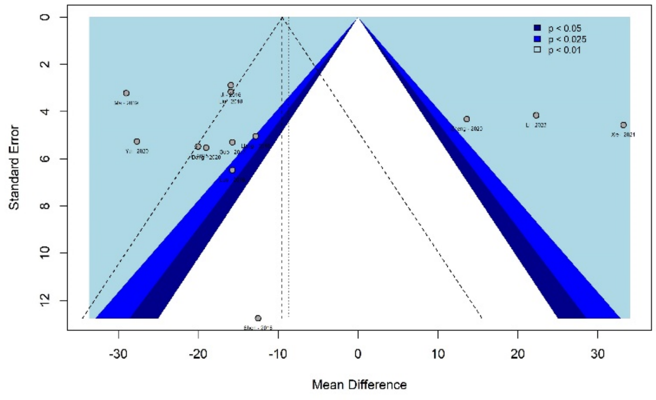


*Operative time*

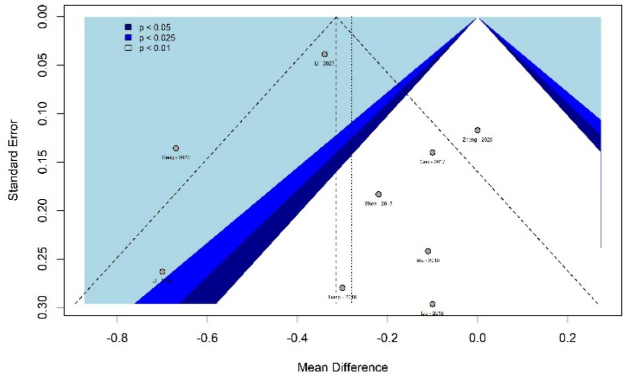


*Time to first flatus*

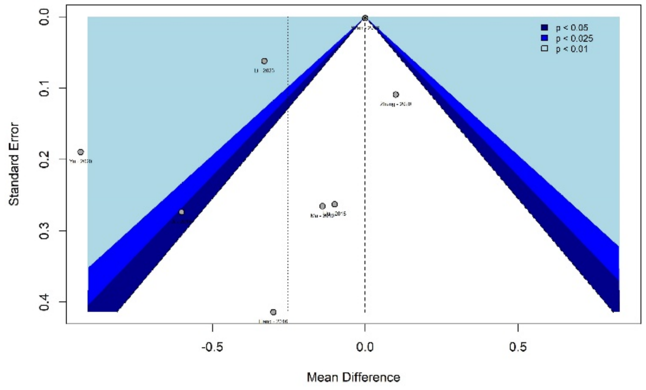


*Time to first liquid intake*

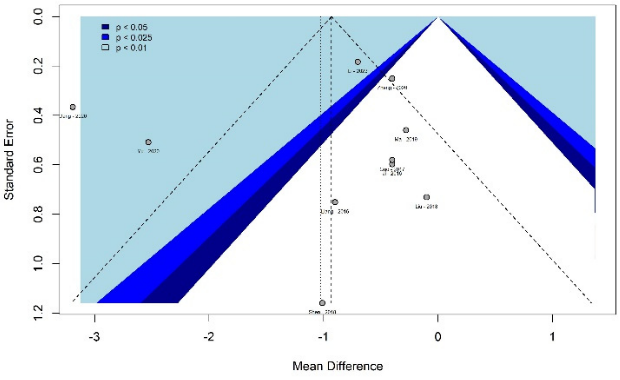


*Length of hospital stay*
