# Supplementary figures and images for: Short-Term Outcomes after D2 Gastrectomy with Complete Mesogastric Excision in Patients with Locally Advanced Gastric Cancer: A Systematic Review and Meta-Analysis of High-Quality Studies
Source: Cancers (Basel). 2023 Dec 31;16(1):199. doi: 10.3390/cancers16010199 (PMC10778561; doi:10.3390/cancers16010199)

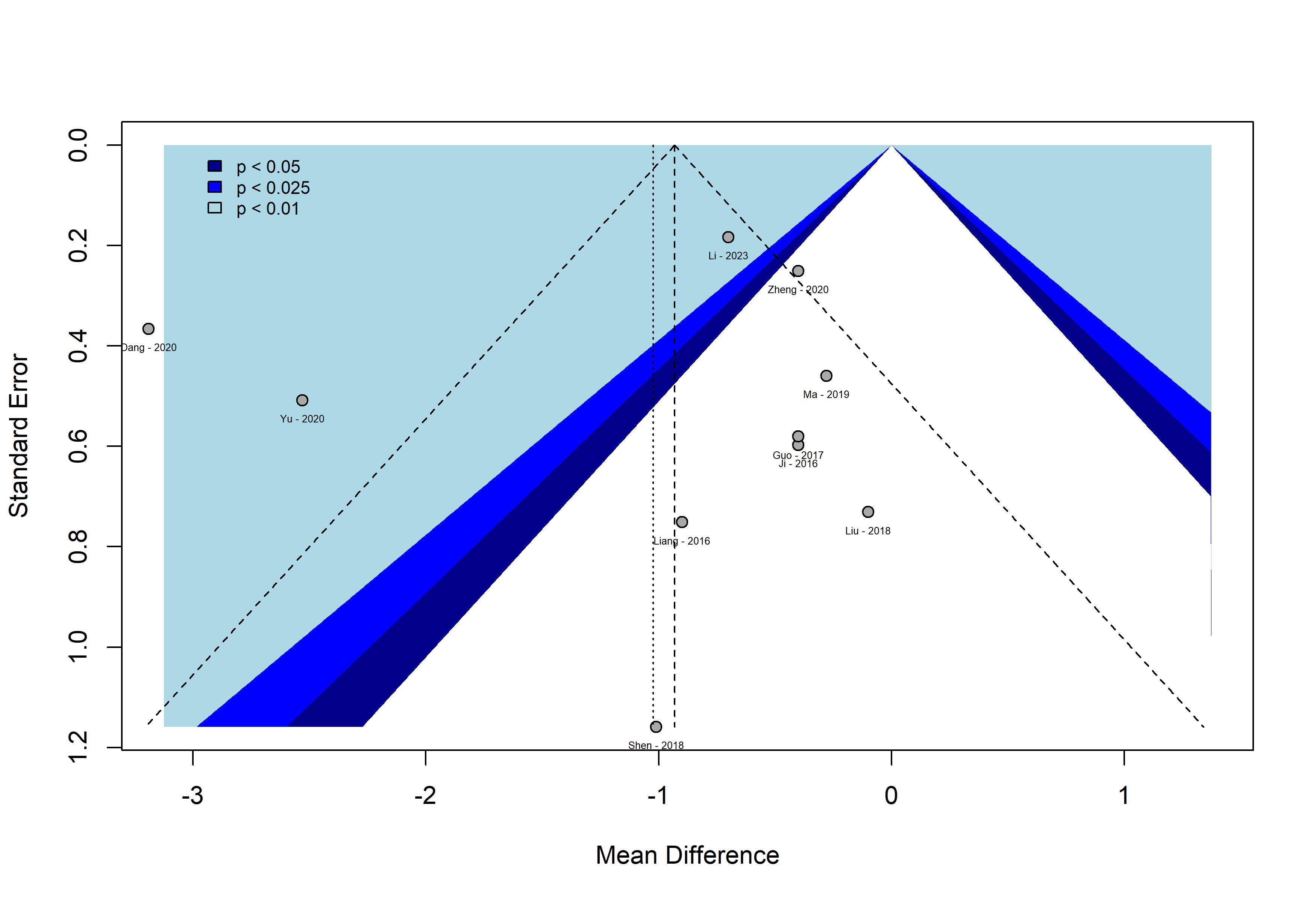

Supplement: Supplementary file 1 [file cancers-16-00199-s001.zip › ce-Funnel Metacont LOS.jpeg]

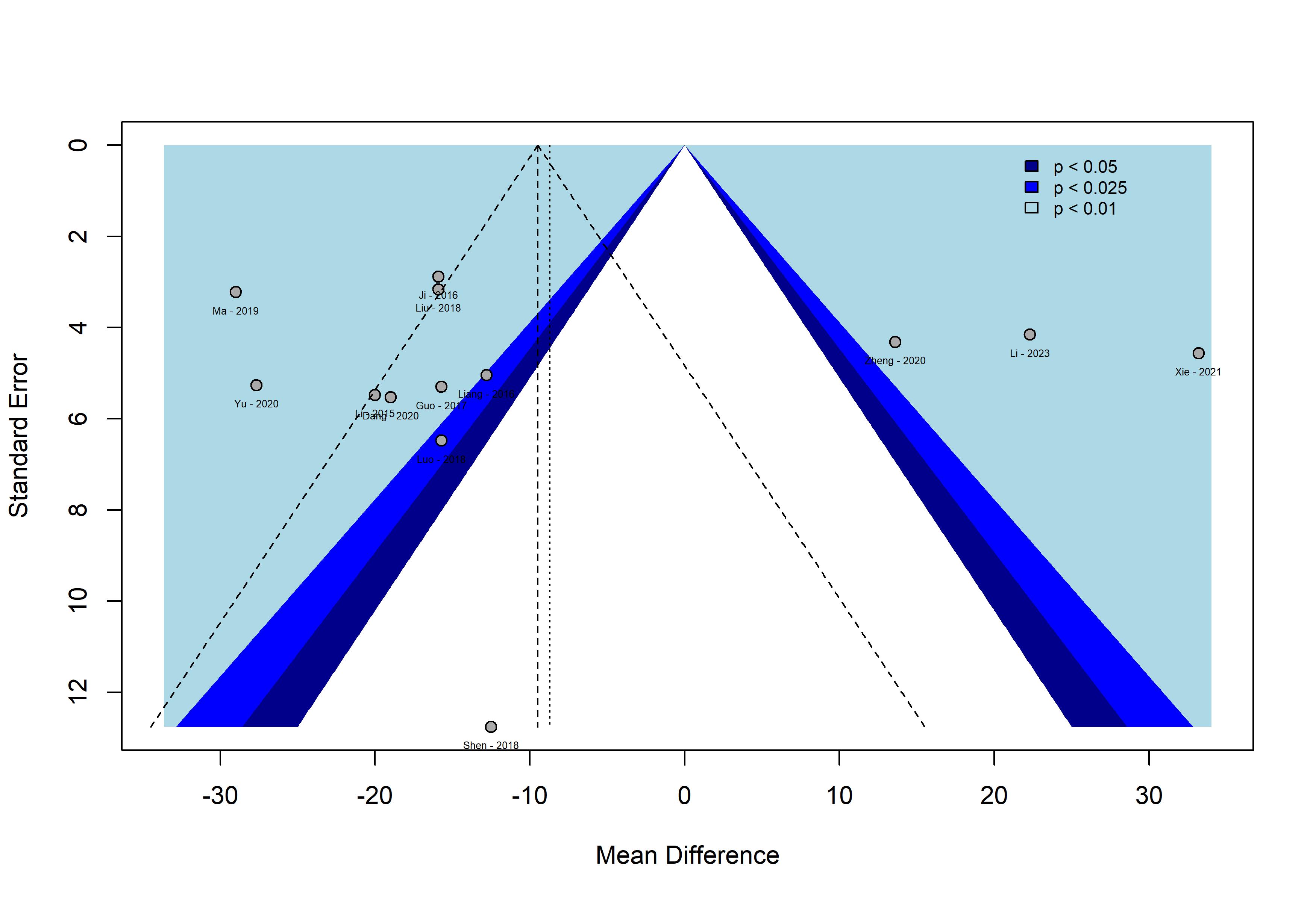

Supplement: Supplementary file 1 [file cancers-16-00199-s001.zip › ce-Funnel Metacont OT.jpeg]

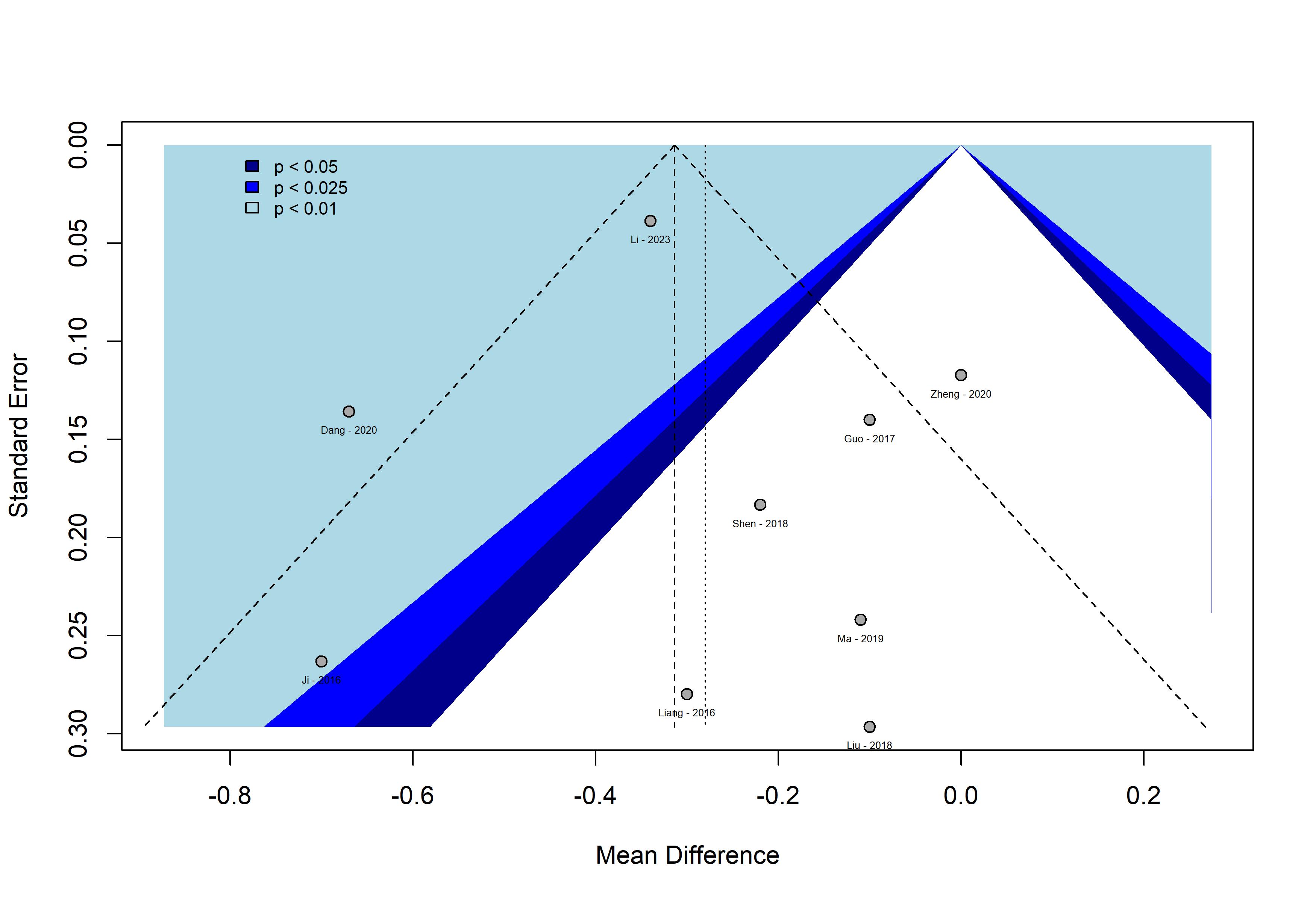

Supplement: Supplementary file 1 [file cancers-16-00199-s001.zip › ce-Funnel Metacont TtFFB.jpeg]

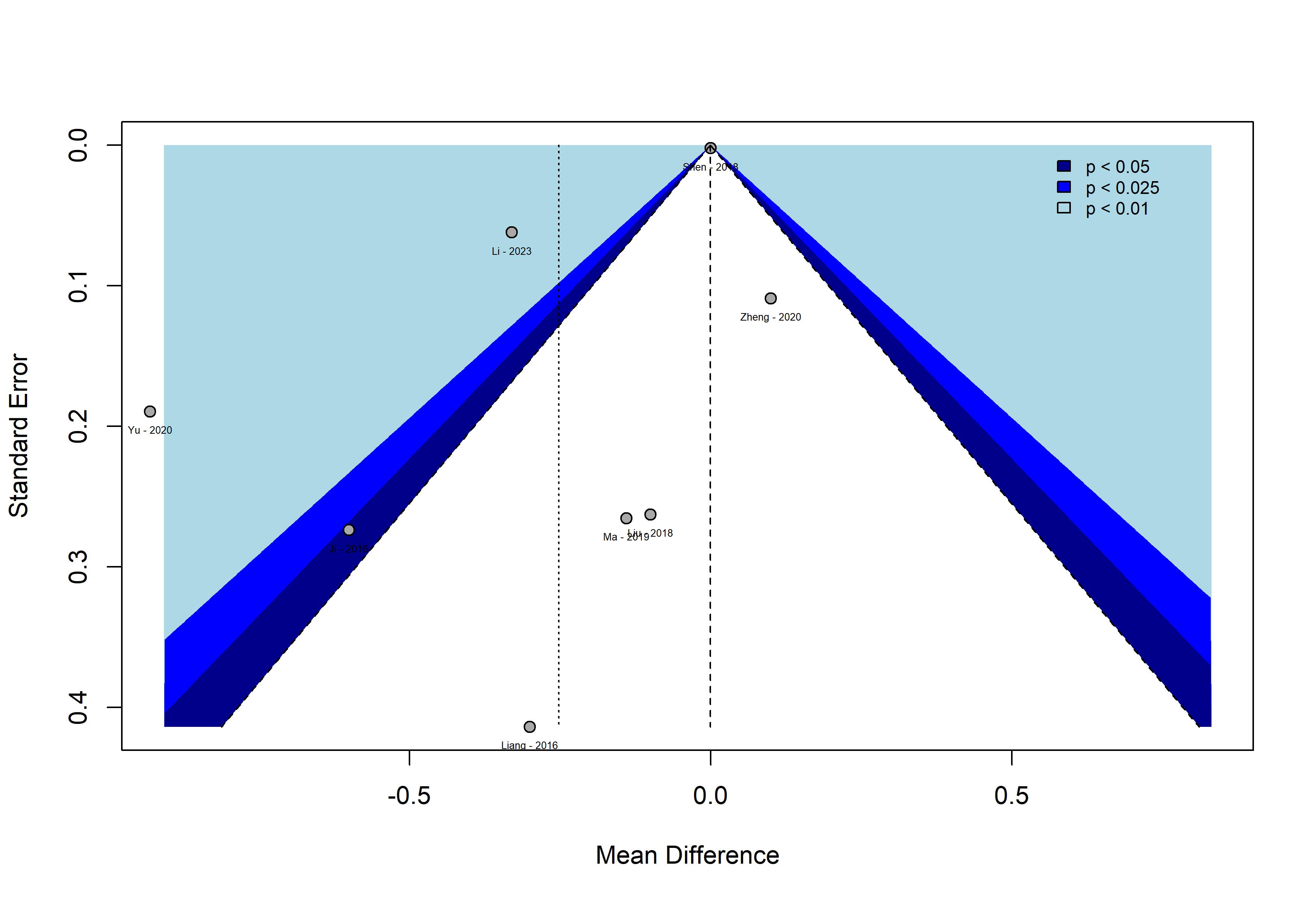

Supplement: Supplementary file 1 [file cancers-16-00199-s001.zip › ce-Funnel Metacont TtFLI.jpeg]

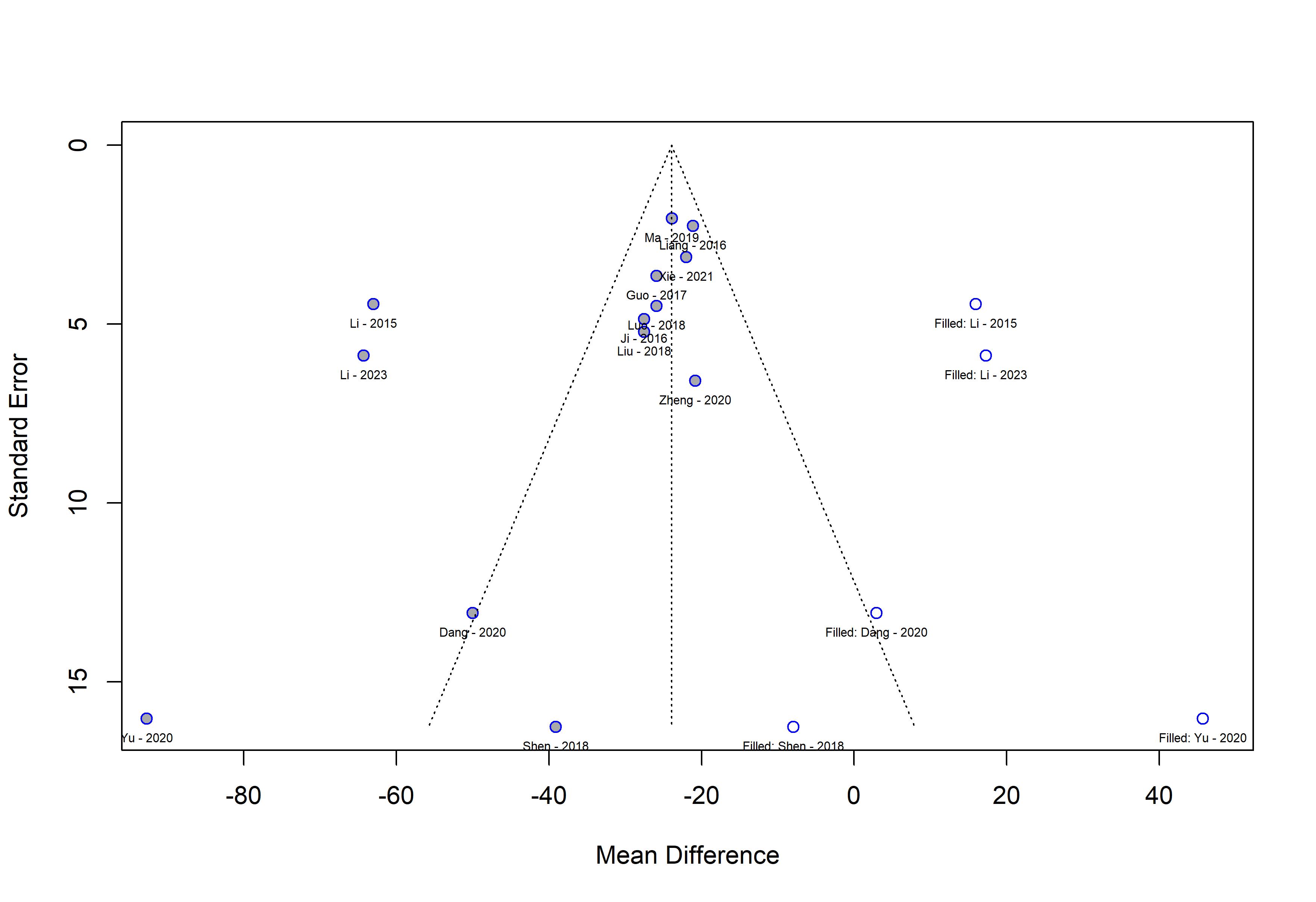

Supplement: Supplementary file 1 [file cancers-16-00199-s001.zip › Funnel Metacont IOB trimfill.jpeg]

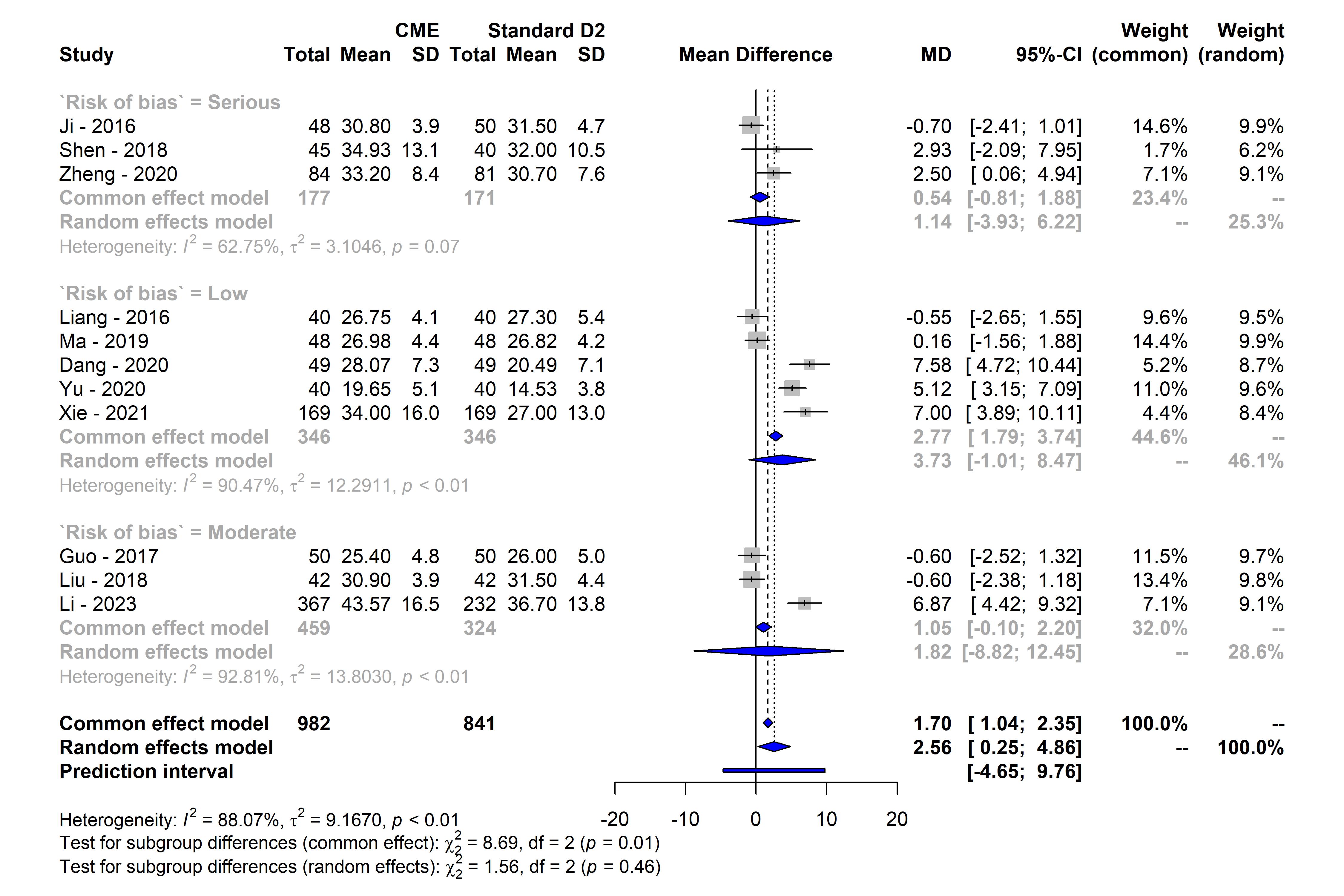

Supplement: Supplementary file 1 [file cancers-16-00199-s001.zip › LNs subgroup Risk of bias.jpeg]

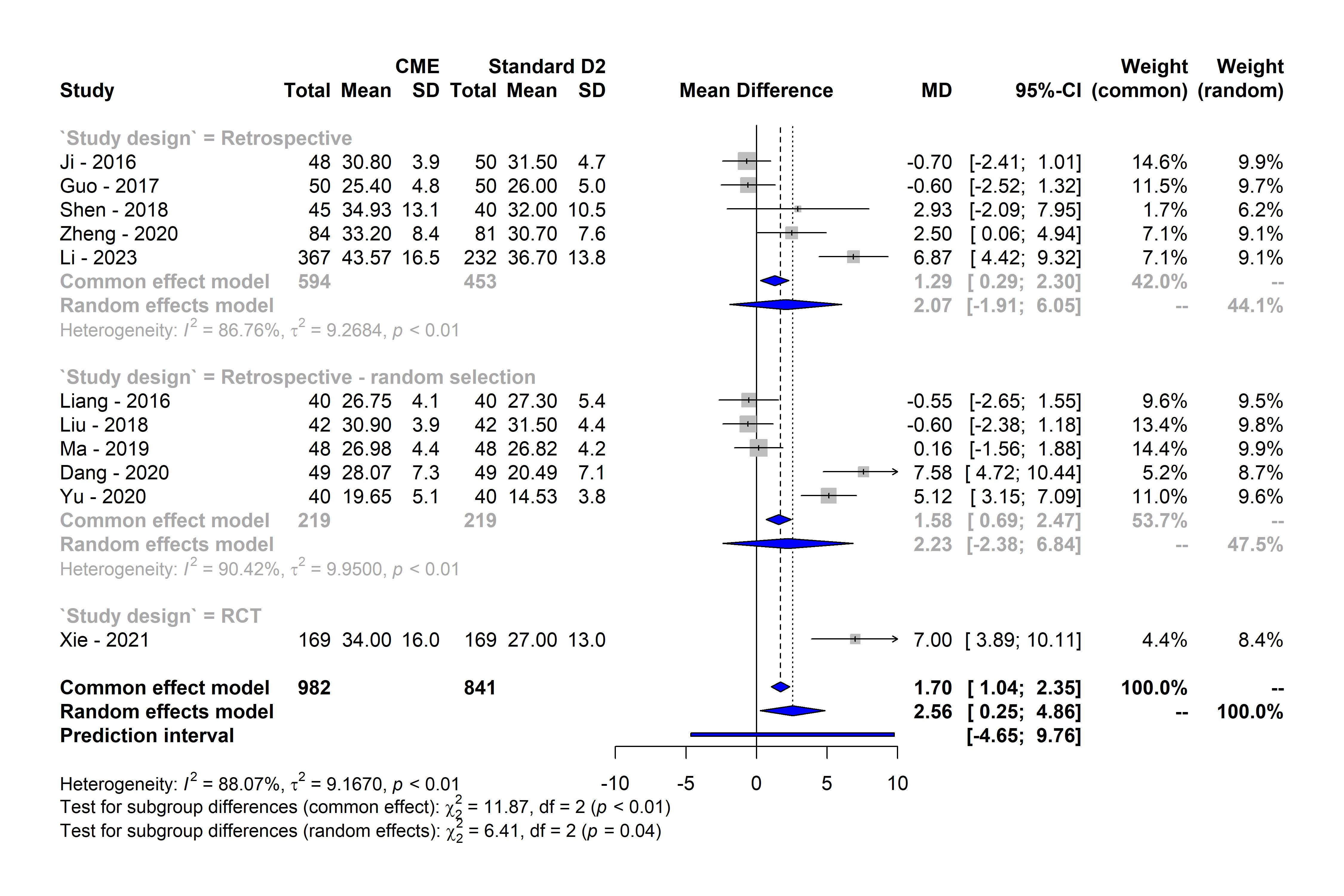

Supplement: Supplementary file 1 [file cancers-16-00199-s001.zip › LNs subgroup study design.jpeg]

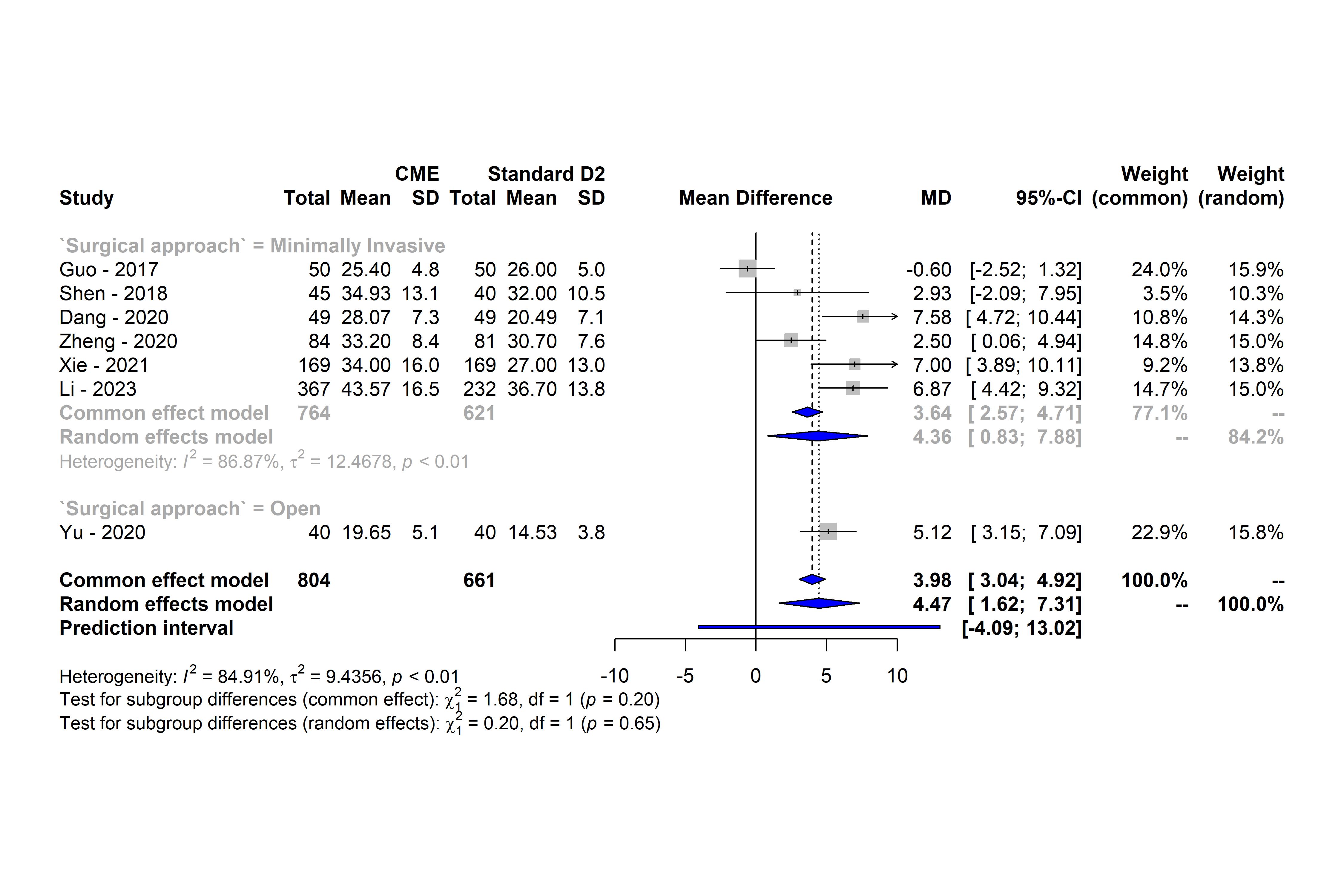

Supplement: Supplementary file 1 [file cancers-16-00199-s001.zip › LNs subgroup surgical approach.jpeg]

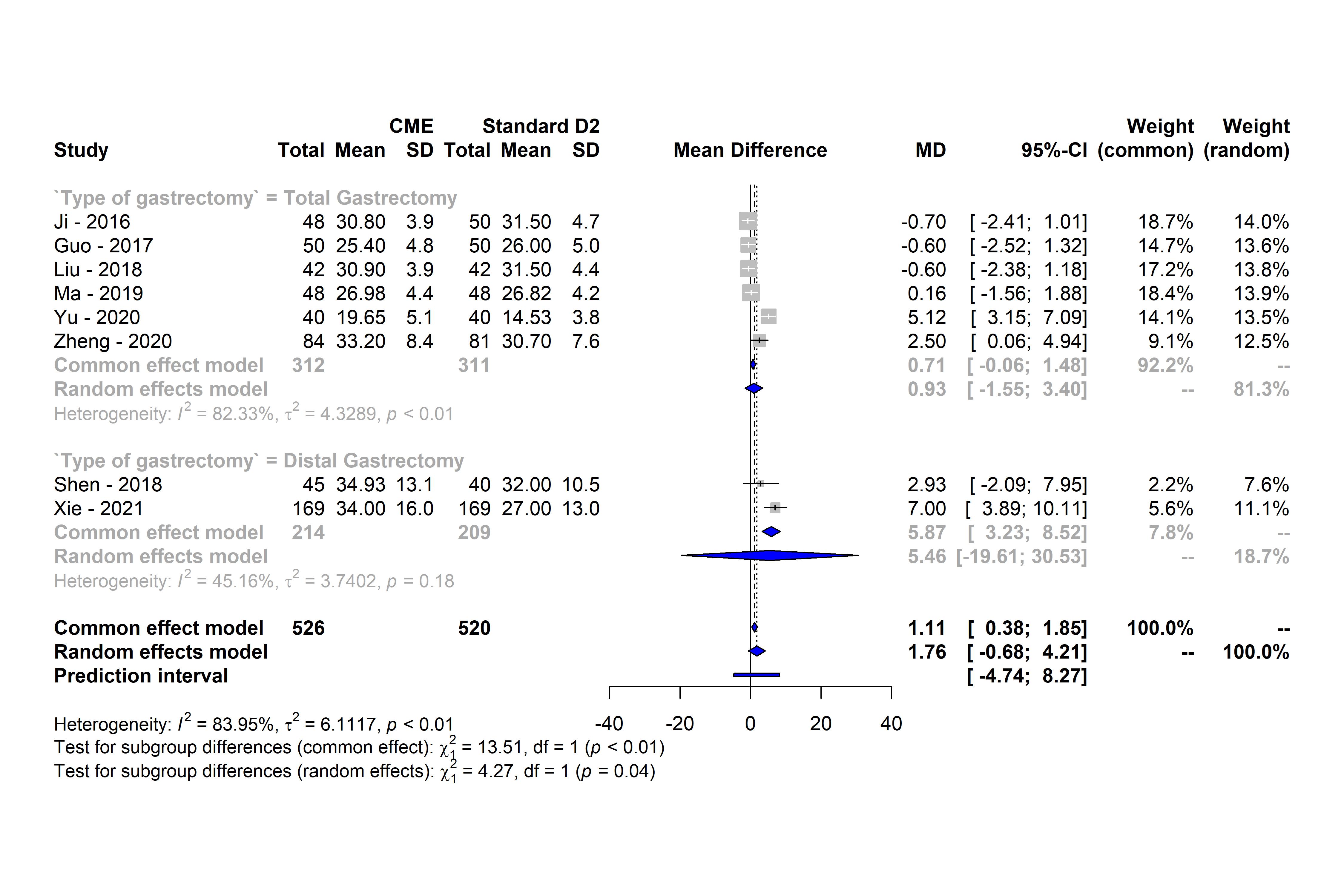

Supplement: Supplementary file 1 [file cancers-16-00199-s001.zip › LNs subgroup type of gastrectomy.jpeg]

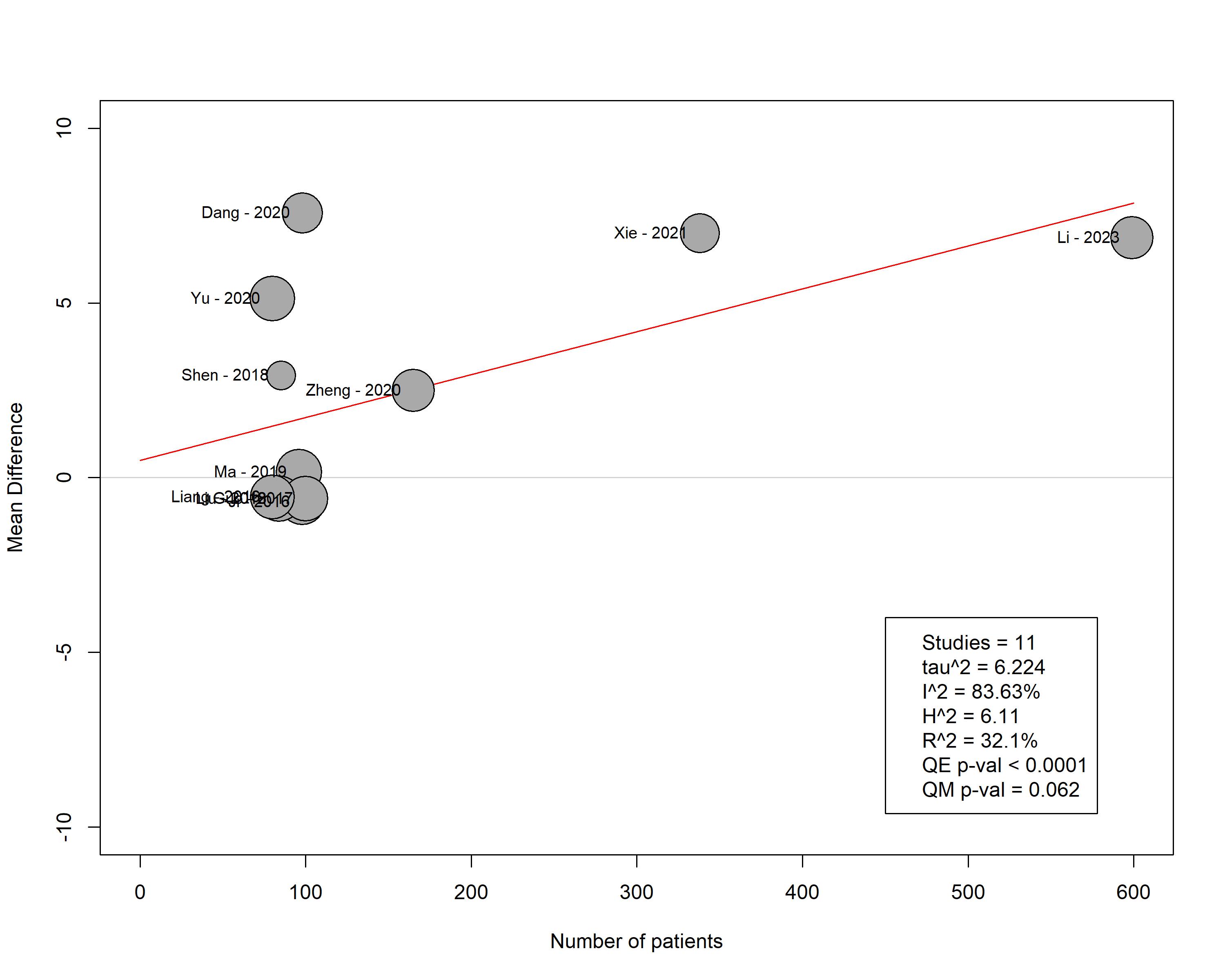

Supplement: Supplementary file 1 [file cancers-16-00199-s001.zip › Total patients.jpeg]

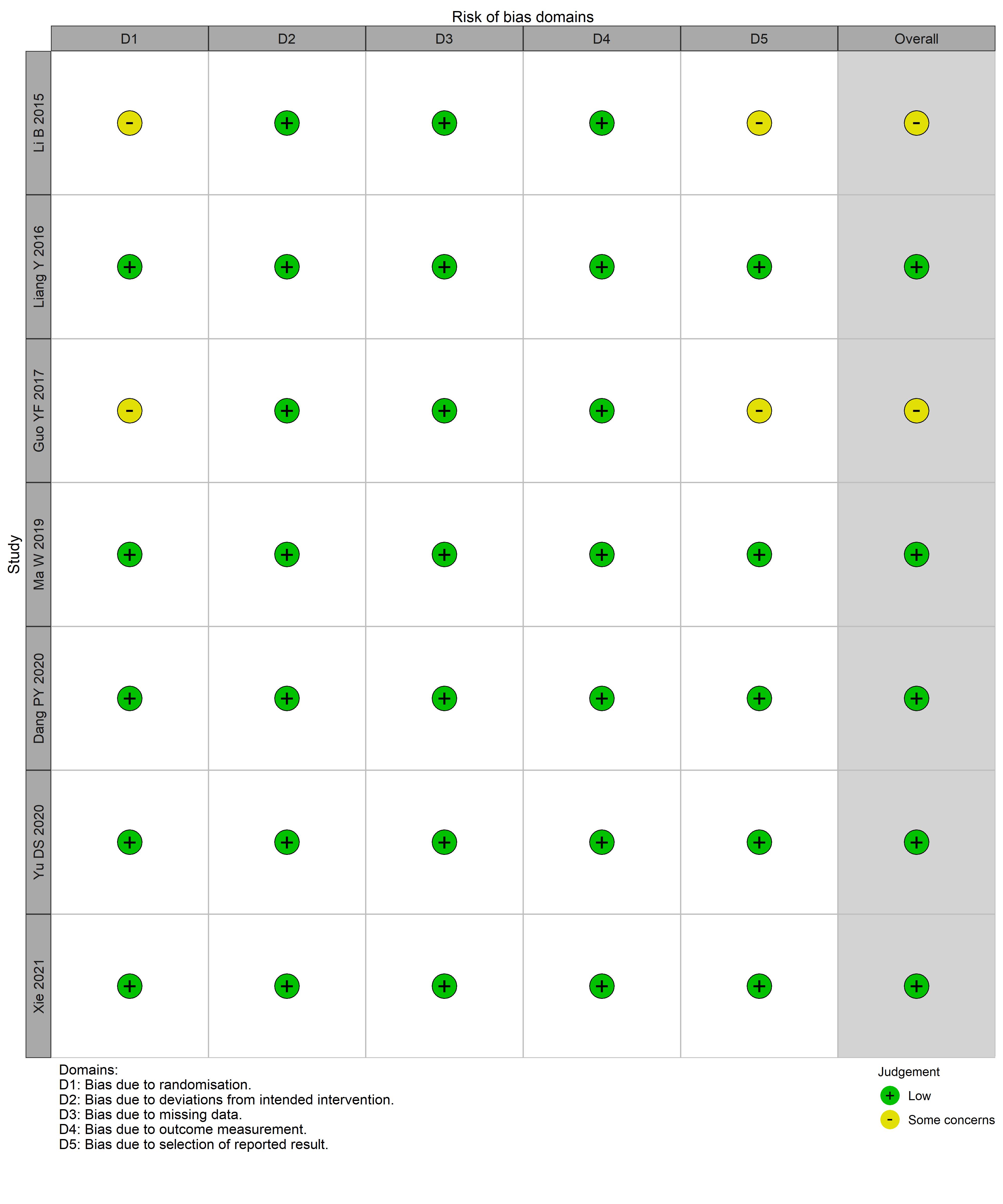

Supplement: Supplementary file 1 [file cancers-16-00199-s001.zip › Traffic light plot RoB2.jpeg]

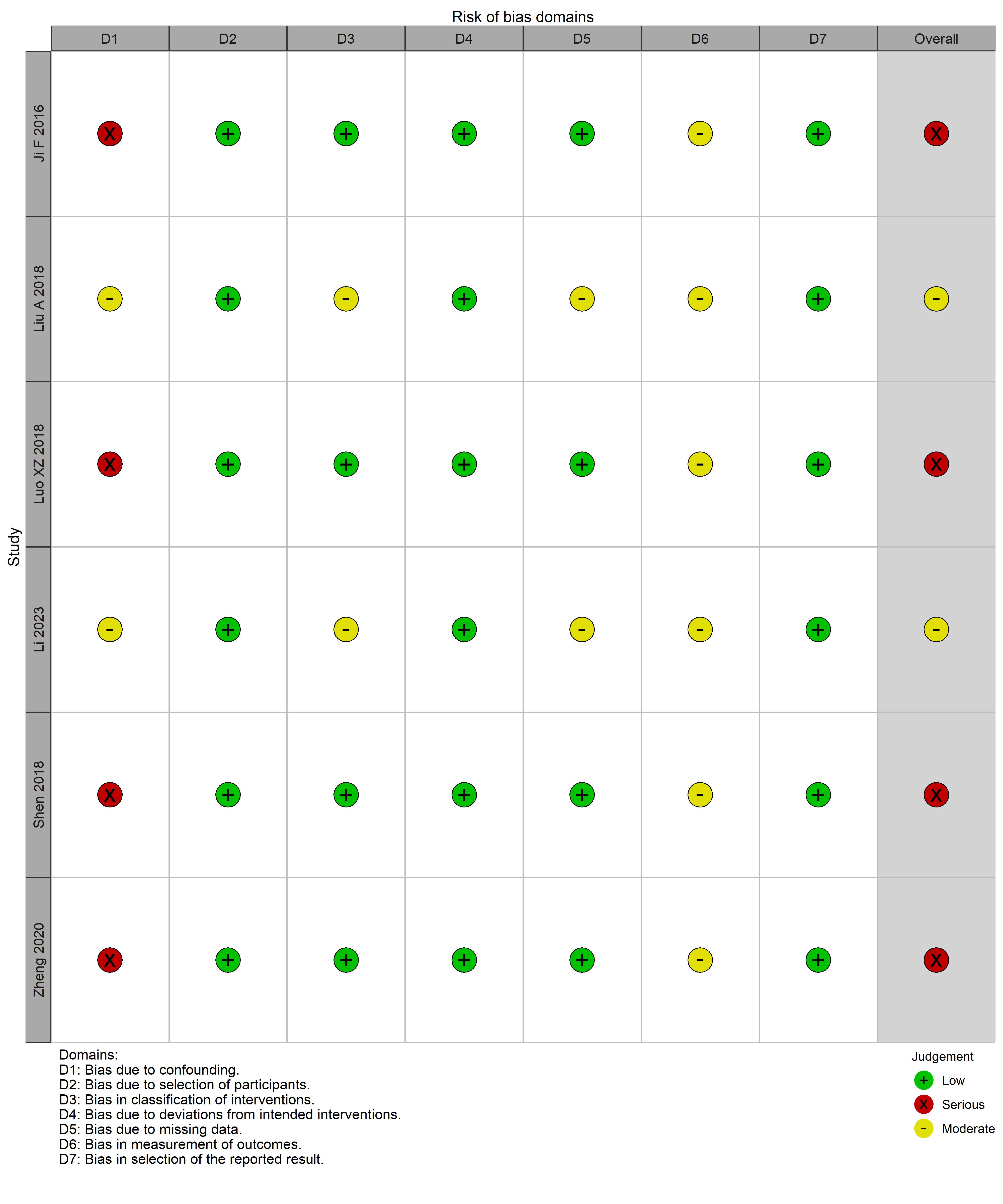

Supplement: Supplementary file 1 [file cancers-16-00199-s001.zip › Traffic light plot ROBINS-I.jpeg]

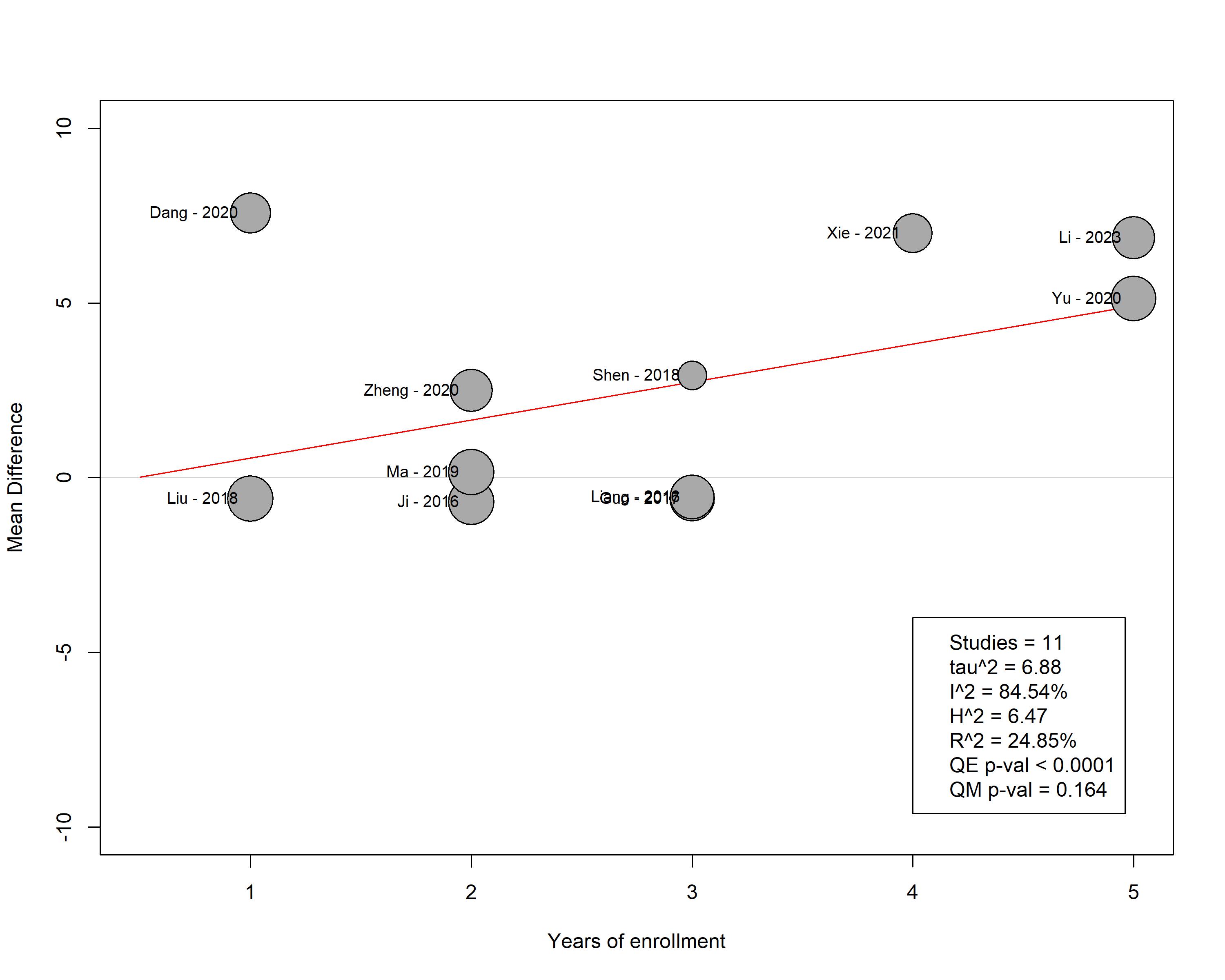

Supplement: Supplementary file 1 [file cancers-16-00199-s001.zip › Years_enrollment.jpeg]

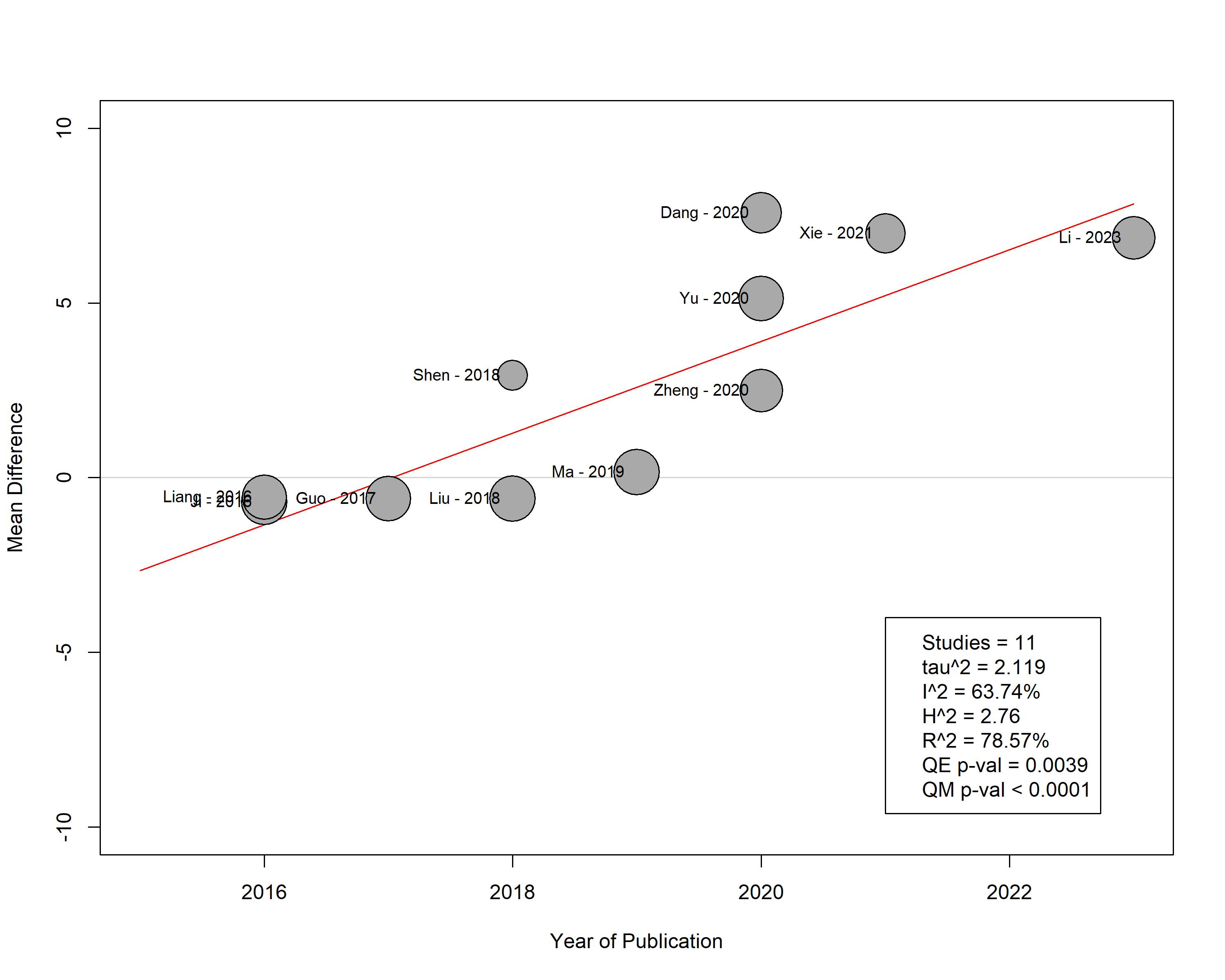

Supplement: Supplementary file 1 [file cancers-16-00199-s001.zip › Year_pub.jpeg]
